# A core outcome set for clinical trials in whiplash-associated disorders (WAD): a study protocol

**DOI:** 10.1186/s13063-018-3019-3

**Published:** 2018-11-19

**Authors:** Annick Maujean, Linda Carroll, Michele Curatolo, James Elliott, Helge Kasch, David Walton, Michele Sterling

**Affiliations:** 1 Recover Injury Research Centre, NHMRC Centre of Research Excellence in Recovery Following Road Traffic Injuries, The University of Queensland, Herston, QLD Australia; 2 School of Public Health, University of Alberta, Edmonton, AB Canada; 3 Department of Anesthesiology and Pain Medicine, University of Washington, Box 356540, 1959 NE Pacific Street, Seattle, WA 98195-6540 USA; 4 Feinberg School of Medicine, Department of Physical Therapy and Human Movement Sciences, Northwestern University, Chicago, IL 60611 USA; 5 Faculty of Health Sciences and Northern Sydney Local Health District, The University of Sydney, Sydney, NSW 2065 Australia; 6 Research Department, Spinal Cord Injury Centre of Western Denmark, Viborg, Denmark; 7 Department of Neurology, Regional Hospital of Viborg, Viborg, Denmark; 8 Institute of Clinical Medicine, Aarhus University, Aarhus, Denmark; 9 School of Physical Therapy, Western University, London, ON Canada

**Keywords:** Outcome measures, Core outcome set, Clinical trials, Whiplash-associated disorders (WAD), Pain

## Abstract

**Background:**

Whiplash-associated disorders (WAD) as a consequence of a motor vehicle crash are a costly health burden in Western societies. Up to 50% of injured people do not fully recover. There have been numerous clinical trials and cohort studies conducted for WAD with many varied outcome measures used, making data pooling difficult and hindering meta-analysis. These issues could be addressed through the development of a core outcome set (COS) that should be included in all clinical trials for WAD. The purpose of this project is to develop and disseminate a COS for clinical trials in WAD.

**Methods/design:**

An international Steering Committee was formed to initiate and support the development of this COS. The project will comprise five phases: (1) a comprehensive review of core outcome domains used in clinical trials in WAD, (2) an international Delphi survey including individuals with WAD, health care providers, clinical researchers and insurance personnel to define the core outcome domains, (3) a meeting of relevant stakeholders to reach consensus regarding the final core outcome domains, (4) identification and evaluation of instruments used to measure the final core outcome domains, and (5) a consensus meeting to agree on the core outcome measurement instruments to be used.

**Discussion:**

The aim of this proposal is to complete a five-stage process to develop a COS for all clinical trials in WAD. An implementation strategy will also be proposed.

## Background

The Global Burden of Disease study identified back and neck pain as the leading global causes of disability in 2015 [1]. Whiplash-associated disorders (WAD) are a costly health burden in Western societies [2] and, given that WAD incur greater disability than non-traumatic neck pain [3], they contribute to much of this burden.

There have been numerous cohort studies and randomised clinical trials conducted for WAD with many and varied outcome measures used, making it difficult to effectively pool data and usually precluding meta-analysis. Recent systematic reviews of prognostic indicators for poor recovery note the inconsistent use of validated outcome measures as a shortcoming of current research [4, 5] with Walton et al. querying the appropriateness of data pooling for this reason [5]. Systematic reviews of treatment have suffered the same problems. Authors of these reviews also call for more consistent use of validated outcomes in clinical trials [6, 7].

These issues could be addressed through the development of a core outcome set (COS). A COS represents an agreed set of outcomes that should be measured and reported, as a minimum, in all clinical trials for specific health conditions [8]. The COS does not mean that outcome measures should be limited to only those in the COS as researchers may need to include additional outcomes appropriate to their trial. It rather mandates collection and reporting of the COS alongside any other outcomes of interest [8]. The first step in the development of a COS is to agree upon the core outcome domains that should be measured [9]. Subsequent steps include evaluation of outcome measure instruments that measure the core domains and finally recommendation of the COS [9].

Recommendations on core outcome domains and measures have been developed for various conditions including the Outcome Measures in Rheumatology (OMERACT) initiative in arthritis [10], the Initiative on Methods Measurement and Pain Assessment in Clinical Trials (IMMPACT) for pain in general [11, 12] and for low-back pain [13]. The International Classification of Function, Disability, and Health (ICF) has recognised the importance of core sets of constructs (ICF codes) that are most germane to different health conditions and created core sets for multiple health disorders [14, 15]. In the area of whiplash research there are yet to be any developments in this area. Considering the health burden of this condition and its often compensable nature, the use of standardised core outcomes is essential in order to pool data and evaluate the effects of treatments to inform both practice and funding policy. Further, WAD is a unique disorder that is unlikely to be sufficiently served by generic musculoskeletal or pain COSs.

The aim of this study will be to develop a COS recommended for use in all clinical trials for WAD.

## Methods/design

### Definitions of key concepts

The definitions of the key concepts and terms used in the current protocol are adapted from the OMERACT initiative [16] and are presented in Table 1.

**Table 1:** Definitions of key concepts adapted from the Outcome Measures in Rheumatology (OMERACT) initiative [16]

Key concept	Definition
Health condition	A situation of impaired health
Health intervention	An activity performed by, for, with or on behalf of a client(s) whose purpose is to improve individual or population health, to alter or diagnose the course of a health condition, or to improve functioning
Core area	An aspect of health or a health condition that needs to be measured to appropriately assess the effects of a health intervention
Domain	Component of core area: a concept to be measured, a further specification of an aspect of health, categorised within a core area
Outcome	Any identified result in a domain arising from exposure to a causal factor or a health intervention
Measurement instrument	A tool to measure a quality or quantity of a variable, in this context a domain or a contextual factor
Outcome measurement instrument	A measurement instrument chosen to assess outcome
Core domain set	For studies of health interventions, the minimum set of domains necessary to adequately cover all core areas, i.e. fully measure all relevant concepts of a specific health condition within a specified setting
Core outcome Measurement set	The minimum set of outcome measurement instruments that must be administered in each intervention study of a certain health condition within a specified setting to adequately cover a corresponding core domain set. Describes how to measure
Setting (scope)	The set of factors that describes the studies and circumstances to which the core outcome measurement set will apply. This is determined by the study questions and includes the health condition(s), target population, interventions, etc.
Contextual factor	Variable that is not an outcome of the study, but needs to be recognised (and measured) to understand the study results. This includes potential confounders and effect modifiers

### Establishing an International Steering Committee

An International Steering Committee was formed to initiate and support the development of this COS. The Committee includes seven members from Australia, North America, and Europe. Members have expertise in the areas of clinical research in the area of WAD (all), clinical practice (MS, HK, MC) and clinimetrics (DW, AM). The co-ordination of the day-to-day management of the project will be undertaken by the Queensland, Australia team (MS, AM). These researchers will co-ordinate the initial review of the literature and the Delphi survey. The remaining members of the Steering Committee will advise on the process via email and/or Skype, as required.

### Scope of the core outcome set

The COS will be recommended for use to measure the efficacy or effectiveness of any health intervention in clinical trials for patients with WAD. WAD are defined as the clinical manifestations (e.g. neck pain, headache, dizziness) arising from an acceleration-deceleration mechanism of energy transfer to the neck. It may result from rear-end or side-impact motor vehicle collisions, but can also occur after other injury mechanisms [17]. The Quebec Task Force (QTF) classification system is the most commonly used system [17] to classify patients with WAD. Under the QTF system, WAD is classified based on the presence of specific symptoms and physical signs. The QTF grades are as follows: grade 1 – Neck complaint of pain, stiffness or tenderness only with no physical sign; grade II – Neck complaint and musculoskeletal sign(s) including decreased range of motion and point of tenderness; grade III – Neck complaint and neurological sign(s) such as decreased or absent tendon reflexes, weakness and sensory deficits; grade IV – Neck complaint and fracture or dislocation [17]. WAD grades I–III will be the focus of the development of this COS.

### Identification of existing knowledge

Prior to developing the protocol for the current project, a search was conducted to identify initiatives that could potentially overlap with this study. The Initiative on Methods, Measurement, and Pain Assessment in Clinical Trials (IMMPACT) [11, 12] comprises a core set of outcome domains and measurement tools to use in clinical trials for chronic pain and partially overlaps with the present COS. However, the IMMPACT COS is very broad, being recommended for pain in general. WAD is a very specific condition that causes significant personal and economic burden and is commonly managed within a compensation scheme of some kind. Further, the neck is a highly complex structure and WAD more than many other conditions also tends to manifest other non-pain-related symptoms such as cognitive difficulties, dizziness, and visual disturbances among other symptoms [18]. This justifies the development of a COS specific to WAD as has been done for other conditions such as low-back pain [13] and shoulder pain [19] among others.

### Stakeholder involvement

COS methodologists recommend that multiple stakeholder groups should be involved in the development and consensus process of a COS [8]. For this reason, four main groups of stakeholders who are the most relevant for the development of a WAD COS will be included:

1. Clinical researchers: this group will comprise of professionals working in various research fields relevant to WAD (e.g. physiotherapy, psychology, pain medicine, and rehabilitation medicine), methodologists, and health economists who currently work predominantly as researchers. Criteria for researchers involved in this project will include authorship of at least two scientific articles on clinical research for WAD
2. Health care providers: this group will comprise professionals from various relevant disciplines such as general practitioners, physiotherapists, chiropractors, and psychologists. To be involved in the current project, health care providers will be required to have clinical experience in the management of individuals with a diagnosis of WAD
3. Insurance personnel: these are professionals involved in the management of Compulsory Third Party or workers’ compensation claims, either in a regulatory capacity or from an insurance company
4. Patients: this group will consist of people who have either previously experienced or are currently experiencing WAD. They will be recruited from a number of sources including: physiotherapy and general medical practices, respondents to advertisements (flyers, newspaper, newsletter, and the Internet) as well as participants involved in previous research studies. It is important to involve patients when developing the COS to gain their perspective of living with a whiplash injury as this may differ from those of the other three stakeholders group

### Conceptual framework

The OMERACT Filter 2.0 initiative provides a useful conceptual framework for the development of a COS for WAD. This framework consists of three core areas that cover the *impact of health condition* (‘Death’, ‘Life Impact’, and ‘Resource Use/Economic Impact’) and which describe *pathophysiological manifestations*. OMERACT recommends the inclusion of at least one domain from each core area in every COS [20]. These domains can be generic or more specific (e.g. disease-specific, time-specific). The core areas and suggestions for domain specification are outlined in the following sections. The OMERACT Filter 2.0 framework has been recently used in the development of COSs for other musculoskeletal conditions [13, 19].

#### Impact of health conditions

Under the core area ‘Life Impact’, OMERACT recommends when developing a COS that both the domains of International Classification of Functioning, Disability, and Health (ICF) (e.g. ICF domains: activity and participation) [16] and the domains pertaining to the concept of health-related quality of life (e.g. loss of ability to work, psychosocial impact, and utility) are considered.

The core area ‘Resource Use/Economical Impact’ relates to the economic impact of health conditions on both the individual and society. OMERACT recommends that at least one domain related to this core area (e.g. recording direct costs, general practice and physiotherapy services for treatment of whiplash injury, patients’ lost economic productivity due to WAD) is included in clinical trials given that increasing health care costs are becoming a challenge for most countries [16].

Under the core area ‘Death’, possible domains include generic and disease-specific, namely all cause vs disease-specific mortality, and intervention-specific such as death due to surgery. However, in health conditions where death rarely occurs during a clinical trial such as in a trial of conservative treatments for individuals with WAD, this area can be covered in the core set by simply including a report of any deaths (or lack thereof) during the trial.

#### Pathophysiological manifestations

According to the OMERACT initiative, the assessment of pathophysiological manifestations is critical to measure whether or not the intervention specifically targets the pathophysiology/functional change of the health condition. Domains related to this core area may include reversible manifestations (including modifiable risk factors and actual indicators of ill health), and irreversible manifestations (including unmodifiable risk factors and damage) [16].

### Research methods

This project will comprise five phases. Phase 1 will generate a list of core domains used in clinical research of patients with WAD. Phase 2 will include an international Delphi study involving relevant stakeholders including patients with WAD, clinicians, clinical researchers and insurance personnel. Phase 3 will involve a consensus meeting with relevant stakeholders with the aim to reach consensus regarding the final core outcome domains to be used in all clinical trials in a research or clinical setting. Phase 4 will aim to identify instruments used to measure the final core domains selected in phase 3. Phase 5 will involve a consensus meeting to agree on the core outcome measurement instruments to be used.

#### Phase 1 – Identify core outcome domains used in the research of WAD

The aim of phase 1 will be to identify domains currently used in the research of WAD, either in measuring outcomes of clinical trials or clinical studies. Four databases (Cochrane Library, CINAHL, PubMed, and Embase) will be searched for clinical trials, randomised controlled trials and systematic reviews relating to WAD from inception until the date of the searches are conducted.

All references will be exported to an Endnote library and sorted by the domains included as outcome measures. Two reviewers will evaluate the studies for inclusion and will extract relevant outcome domains used. The domains will then be classified as per the core areas defined in the OMERACT Framework. Once identified, the listed core domains will then be critically reviewed by the international Steering Committee. Committee members can indicate additional domains or make comment on identified domains in terms of aggregation. The final list of potential outcome domains will be used in the Delphi survey.

#### Phase 2 – International Delphi Study

An international Delphi Study will be conducted to reach consensus on the core domains identified in phase 1. The Delphi technique consists of successive surveys that a panel of participants with relevant expertise complete anonymously. The advantage of using such an approach to reach consensus is that participants do not interact directly with each other as would occur with a face-to-face meeting. Therefore, the group of participants is not influenced by the views of a few dominant individuals [19]. In addition, this approach provides the opportunity to include participants from many countries because the survey is administered online. The current study will comprise of a three-round Delphi survey.

Participants in the Delphi surveys will come from the four identified stakeholder groups, i.e. clinical researchers, health care providers, insurance personnel, and patients. Based on previous research a minimum of 80 participants will be required for each round [13, 19] and to achieve this, 200 participants will be initially invited, allowing for a 60% non-response rate. The criteria for inclusion for each stakeholder group will be as outlined earlier. The final list of participants will remain confidential to all except for the local project team.

##### Delphi round 1

In round 1, participants will be provided with information about the aims of the study and the survey. The Delphi survey will include the list of all domains identified in phase 1 and each domain will be accompanied by a brief description to facilitate common understanding of the construct. A text box will be provided for participants to list any additional domains that they believe are important and had not been included. In addition, participants will be asked to provide their opinion about the number of core domains that they believe should be included in the COS and whether there are any domains listed in the survey that they think could be combined. Participants will be instructed to rate the importance of each domain on a Likert scale from 1 to 9, with 1 to 3 labelled ‘not important’, 4 to 6 ‘important but not critical’, 7 to 9 ‘critical’. They will be given 2 weeks to respond. For those who do not respond within the 2-week period, a reminder email will be sent, allowing an additional week for participants to complete the survey.

The frequencies for the response options on the importance of each domain across all participants and for each group of stakeholders will be calculated. After round 1, the number of domains to be included in round 2 will be reduced in order to prevent participant burden. The criteria for inclusion in round 2 will be based on that suggested by the COMET Handbook [8]. Items rated 7 to 9 by 50% or more participants and 1 to 3 by no more than 15% of participants in at least one stakeholder group will be retained.

##### Delphi round 2

Results of round 1 will be emailed to all Delphi participants together with the round-2 survey. Any suggestions from respondents for inclusion of additional domains or to combine domains will be discussed by the Steering Committee before inclusion in round 2. The overall process for round 2 will be similar to round 1. Participants will have 2 weeks to respond to the survey and for those who have not responded within the 2 weeks allocated, a reminder email will be sent to those participants and they will be allowed an additional week to respond. Participants will be asked to re-evaluate the importance of all domains that reached consensus in round 1 and domains suggested by participants that were not included in the initial round. The inclusion of items at the end of round 2 will use stricter cut-off criteria [8] – retaining items rated between 7 and 9 by over 70% of respondents and 1 to 3 by less than 15% by at least one stakeholder group. This process of using stricter criteria through the Delphi rounds has been recommended as a way to reduce the likelihood of dropping outcomes that may have been rated more highly in subsequent rounds, had the participants been provided with feedback [8]. At completion of round 2, frequencies for the response options will be calculated for the whole group of stakeholders and separately for each group of stakeholders to establish whether there are discrepancies between the groups.

### Delphi round 3

Results of round 2 will be emailed to all Delphi participants together with the round-3 survey. Participants will again be invited to participate in round 3 and the overall process will be similar to the previous two rounds. At completion of round 3, frequencies for the response options will be calculated for the whole group and separately for each group of stakeholders. Consensus that a domain should be included in the core outcome domain set is defined as the domain having a rating of 7–9 by ≥ 70% and 1–3 by ≤ 15% by all four stakeholder groups or rated as 7–9 by ≥ 90% of one group [21].

#### Phase 3 – Final decisions

The project team will present the results of the Delphi survey for all rounds at a face-to-face consensus meeting. Attendees at this meeting will include the international Steering Committee, clinical researchers, health care providers, insurance and patient representatives. The intention is to hold this meeting at a time close to a relevant international conference to maximise participation. The aim of the meeting is to agree on the final core outcome domain set. Any discrepancies or concerns (for example, between different stakeholder groups) will be discussed. If a vote is required, then greater than 50% support from members of the Steering Committee will be required for an outcome domain to be included as part of the COS.

### Phase 4 – Selection of instruments for each core outcome domain

Following completion of the identification of core outcome domains (phases 1–3), the Steering Committee will identify a preliminary set of core outcome measures for the core domains informed by (1) measures frequently used in clinical trials for WAD and (2) published systematic reviews. For core domains with limited information pertaining to outcome measures, systematic reviews will be conducted by members of the Steering Committee. The protocols for these systematic reviews will be registered at Prospero. If there are insufficient studies in WAD for measurement of a particular domain, studies of other relevant conditions (e.g. non-traumatic neck pain) will be included.

The identified outcome measures will be evaluated according to the nine measurement properties identified by the COnsensus-based Standards for the selection of health status Measurement INstruments (COSMIN) initiative [22]: Internal consistency, reliability, measurement error, content validity, structural validity, hypotheses testing, cross-cultural validity, criterion validity, and responsiveness.

#### Phase 5

All findings from this process will be documented and presented to the meeting attendees who participated in the phase 3 meeting, again in a face-to-face format. The findings will be discussed and if voting is required, the criteria for consensus will be similar to the process adopted by Kaiser et al. (2015) [23]. That is, only outcome measures that obtained 70% or more ‘Yes’ vote and 20% or less ‘No’ vote will be included into the final COS to be used across WAD clinical trials.

### Dissemination of the COS recommendations

A dissemination plan similar to the one outlined in Gagnier et al. [19] will be used to optimise the implementation of the recommended COS for the WAD trial. The findings will be disseminated to targeted audiences such as clinical researchers working in various fields relevant to WAD (e.g. physiotherapy, psychology, rehabilitation) and health care providers (e.g. general practitioners, physiotherapists, and chiropractors) primarily via publications and international conferences. In addition, the level of interest that the recommended COS generates among clinicians and researchers will be monitored.

#### Publications

The first step will be to publish the protocol for the development of the COS. At completion of phase 3, the findings of the Delphi survey and the core outcome domains that should be included in all WAD trials (phases 2 and 3) will be submitted for publication. At conclusion of this project (phase 5), the aim will be to publish a paper detailing the core outcome measurement instruments to be used for each domain in WAD trials. In addition, the recommended COS will be published in the COMET database.

#### International conferences

The findings of this project will also be presented at relevant international conferences across different professional disciplines such as clinical researchers, health care providers, health economists, and policy-makers.

#### Assessment of dissemination strategies

To assess the efficacy of the strategies used for dissemination, the level of interest will be monitored by tracking the number of times that the COS recommendations are accessed, downloaded or cited (via citation trackers). Furthermore, evidence of use of the COS in WAD trials will be monitored by reviewing clinical trial registries.

## Discussion

The main objective of this study is to develop a COS that can be recommended for use in all clinical trials for WAD. The process recommended by COMET [20] and the OMERACT initiative [16] will be used to guide the development of this COS. This includes the establishment of an international Steering Committee, the involvement of a range of stakeholders relevant for the development of a COS for WAD (including patients with who have or are still experiencing WAD), and the conceptual framework recommended by the OMERACT initiative for the development of a COS. The strength of this protocol is that it will follow a well-established process that will enable the development of a strong and meaningful COS for WAD. In addition, the inclusion of a range of stakeholder groups (e.g. patients with WAD, health care providers, researchers, and insurance personnel) and international experts in developing this COS will ensure that it will be relevant across professional disciplines and internationally. Finally, a COS for WAD will enable meaningful comparisons across clinical trials to evaluate treatment efficacy and effectiveness for this population. Furthermore, the assessment of core outcome domains using high standard measurement instruments will help in improving the accuracy of the evaluation of these core domains for WAD and in turn will help improve the development of more effective and targeted treatment interventions for this population.

### Study status

The initial literature review to identify core outcome domains has been completed. Participants for the Delphi surveys have been identified and the first-round survey is being complied.

## Abbreviations

COS: Core outcome set
ICF: International Classification of Function, Disability, and Health
IMMPACT: The Initiative on Methods, Measurement, and Pain Assessment in Clinical Trials
OMERACT: Outcome Measures in Rheumatology
QTF: Quebec Task Force
WAD: Whiplash-associated disorders
